# Repeated CMV Infection in a Heart Transplantation Patient

**DOI:** 10.1155/2012/305920

**Published:** 2012-11-04

**Authors:** Josep Melero-Ferrer, Ignacio J. Sanchez-Lazaro, Amparo Navea-Tejerina, Luis Almenar-Bonet, Marino Blanes-Julia, Luis Martinez-Dolz, Antonio Salvador-Sanz

**Affiliations:** ^1^Unidad de Insuficiencia Cardíaca y Trasplante Servicio de Cardiología, Hospital Universitario y Politécnico La Fe, 46026 Valencia, Spain; ^2^Fundaciónn Oftalmológica del Mediterráneo, 46015 Valencia, Spain; ^3^Unidad de Enfermedades Infeciosas, Servicio de Medicina Interna, Hospital Universitario y Politécnico La Fe, 46026 Valencia, Spain

## Abstract

Infections are one of the leading causes of morbidity and mortality in heart transplantation (HTx). Cytomegalovirus (CMV) is the most common viral infection during the first year after HTx, but it is more unusual after this time. We present the case of a patient who underwent an HTx due to a severe ischemic heart disease. Although the patient did not have a high risk for CMV, infection, he suffered a reactivation during the first year and then up to six more episodes, especially in his eyes. The patient received different treatments against CMV and the immunosuppression was changed several times. Finally, everolimus was introduced instead of cyclosporine, and mycophenolate mofetil was withdrawn. The presented case provides an example of how the immunosupresion plays a key role in some infections in spite of being a suitable antiviral treatment.

## 1. Introduction

Infections are one of the leading causes of morbidity and mortality in heart transplantation (HTx). Among viral infections, cytomegalovirus (CMV) is the most common during the first year. In the past few years, there have been many advances in the prevention, diagnosis, and treatment of the CMV infection, but it is still a matter of concern [1].

 Traditionally, CMV infection was managed with specific antiviral treatment, achieving, in the last years with the introduction of valganciclovir, greater results. Although these treatments are quiet effective, in some cases and despite being the antiviral treatment, some patients develop many infections due to CMV. In these situations arise the possibility of CMV resistance, and second line therapies as foscarnet are used, but it is also possible that the immunosuppressive regimen plays a key role [2].

The case report we show goes on a HTx patient who suffered many CMV infections despite taking many antiviral treatments until the immunosuppressive regimen was switched from a conventional to a more specific one.

## 2. Case Report

We here present the case of a 64-years old male who underwent HTx in 2001 due to a severe ischemic heart disease. OKT3 was the immunosuppressive agent selected for induction while cyclosporine (CsA), azathioprine (AZA), and deflazacort were used for maintenance. Immediate postoperative evolution was favorable. Both donor and recipient had positive CMV serology tests for IgG. During the first year after transplantation, the patient presented three episodes of acute rejection which were managed with steroid boluses and after one of these episodes, an elevation in the CMV viral load was detected. According to our protocol, oral ganciclovir (GCV) was started until the complete suppression of the viral load. There were no more complications in the first year. During the first six months after HTx, levels of CsA were 200–300 ng/mL, and after this date, levels were 100–200 ng/mL. Aza dose was usually 75 mg/day to achieve a leucocyte account of 3000–5000/mm^3^. After the first year, deflazacort dose was 6 mg/day.

Eighteen months after the HTx, the patient suffered an episode of bilateral acute CMV retinitis, treated first with intravenous acyclovir and later with oral acyclovir with a good response.

 Three years after the HTx, and according to our new protocol, AZA was switched to mycophenolate mofetil (MMF) with a standard dose of 1000 mg/12 h. That year, the patient developed a new episode of CMV retinitis in his left eye (SE) which required once more intravenous and oral acyclovir followed by oral valganciclovir (VGV) for three months. Progressive optic atrophy developed and visual acuity was poor in this eye. This time, no viruses were found in blood samples.

Nonetheless, the next year, the patient developed a new episode of loss of visual acuity in his right eye (RE), but that time CMV DNA was detected in blood. In this case, the ocular problem could be relieved with VGC and a reduction in the maintenance immunosuppression. MMF was lowered to 500 mg/12 h (before 1000 mg/12 h) and CsA was lowered to the half.

In January 2010, the patient was admitted because of febrile diarrhea. It did not respond to a standard management that included intravenous fluid support and wide spectrum antibiotherapy. CMV DNA was detected in blood (viral load 525,000 copies/mL). An endoscopy was performed and CMV was present in the duodenum mucosa samples taken. The diarrheic process could be solved again with oral valganciclovir. After this episode, CsA was switched for everolimus (EVL) switched for everolimus (EVL) with levels at the discharge of 4.2 ng/mL.

**Figure 1:**
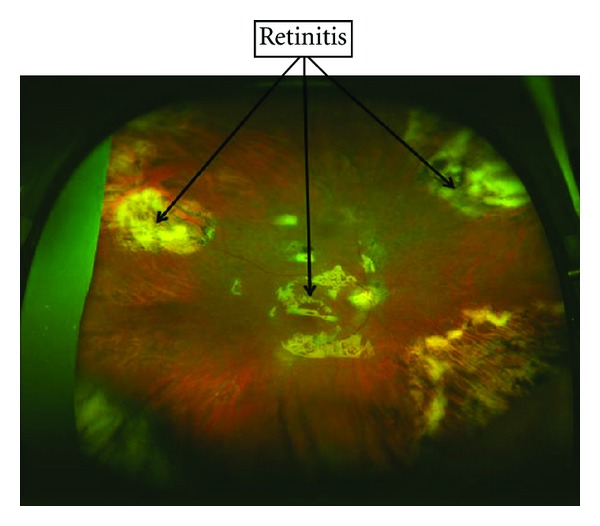


Three months later the patient presented to the Hospital complaining of dyspnea, fever, watery diarrhea, and loss of visual acuity in his RE. Chest X-ray showed a right basal alveolar condensation suggestive of pneumonia and pneumococcal antigen was present in urine samples. Therapy with levofloxacin was initiated with good response. CMV DNA was detected once more in blood (422,000 copies/mL), so this time, suspecting viral resistance to VGC, therapy with foscarnet was prescribed. The patient received intravenous foscarnet for 21 days until complete negativization of CMV load was documented. Ocular examination revealed active chorioretinitis and retinal detachment Figure 1. CMV involvement was confirmed by detecting viral DNA in vitreous humor. This clinical picture could be managed with intraocular administration of foscarnet and vitrectomy to treat the retinal detachment. During the postoperative period, the patient developed a dendritic keratitis in his RE. After complete resolution of the several clinical problems the patient presented, he could be finally discharged under maintenance treatment with intravenous foscarnet (daily for 60 days more) as outpatient. In order to prevent new episodes of CMV infection, MMF was withdrawn.

 Since that last admission, the patient is performing well. The patient receives nowadays EVL + deflazacort as maintenance immunosuppression and VGC in prophylactic doses. CMV viral load has been monitored being all the determinations successively negative. No more severe infectious processes have been documented. Unfortunately patient's outcome is conditioned by the development of severe generalized arteriosclerosis (renal arteries, intermittent claudication, etc.) and his quality of life is very limited by the loss of vision due to previous retinal infections. The evolution of the patient has been resumed in Table 1. 

## 3. Discussion

Cytomegalovirus is a DNA virus that usually affects immunocompromised patients. CMV infection typically occurs after allogenic cells or solid organ transplantation supposing a significant load of morbidity and early mortality. It is also a major factor risk of death and graft loss. The clinical spectrum of this infection includes latent infection, asymptomatic viremia, CMV syndrome, and CMV disease [3].

CMV is a common complication after heart transplantation. The factors most frequently involved in susceptibility to this infection and its severity are donor and recipient CMV serological status, the intensity of immunosuppression, and the type of immunosuppressive agents used [4]. Usually, CMV infection occurs during the first year after HTx. In this time, there are two strategies to prevent CMV infection. The so-called preemptive therapy is based on frequent determinations of viral load to treat the CMV in an asymptomatic phase. On the other hand, some centers give universal prophylaxys (usually VGC) or prophylaxys only to those patients with higher risk (recipient CMV −/donor CMV +). CMV infection frequently presents as an interstitial pneumonia, gastroenteritis, or hepatitis, progressive dyspnea, fever, and diarrhea as the most common symptoms. Rare ocular (retinitis) and encephalitis involvement has been described [5].

Among viruses, CMV is one of those with less resistance develops, but when a CMV strain develops drug resistance, it occurs mainly with GCV/VGC [6]. Compared to GCV, prophylactic treatment with VGC reduces the risk of development of resistant strains due to its better bioavailability which achieves lower viral loads [7]. This patient has experienced episodes of bilateral relapsing CMV retinitis that have lead to an important loss of vision due to the secuelae: optic nerve atrophy in his SE and retinal detachment in his RE. CMV retinitis after HTx has been reported to be progressive and severe and that has been the evolution in this case [8]. 

Risk factors associated with viral resistance are lung transplantation (lung is a major reservoir for CMV), elevated viral load or maintained positive viral load, intensive immunosuppressive regimen, and CMV naïve transplant recipients [9]. CMV resistance must be suspected when a lack of response to a determined antiviral drug is detected (rising viral loads or progressive disease) after two weeks of treatment. To confirm CMV resistance, a viral culture in shell vial and successive detection of specific mutations must be carried out. In most cases, CMV resistance can be successfully managed with a decrease in the immunosuppressive therapy dosage and higher doses of GCV/VGC. In the rest of cases VGC must be switched to foscarnet. Only in a few cases where double mutation is identified in them, combined therapy with both drugs is required [10].

The other key point in preventing CMV infections in transplant recipients is the immunosuppressive regimen that has been chosen. The use of OKT3 during the induction period or for treatment of severe graft rejections has been related to an increase in the rates of viral infections. Moreover, the use of several intravenous steroids courses has a negative impact on the outcome of transplant recipients due to an increase in the number of severe infections [11]. However, both OKT3 and steroids have their maximum influence during the early posttransplantation period. We also have to point that OKT3 is no more available and the new induction therapies such as basiliximab do not have influence in infection rates. As maintenance therapy, most centres use the combination of a calcineurin inhibitor (cyclosporine or tacrolimus) + MMF + steroids. Although some studies point that CsA could favour more viral infections than tacrolimus, the calcineurin inhibitor seems to be neutral in this setting [12]. Years ago, AZA was worldwide switched to MMF in order of the benefits in mortality and rejections with MMF, although these same studies showed a greater CMV infection rate with MMF [13]. More recently, EVL has been associated with fewer viral infections (CMV included) compared to AZA and MMF in renal and HTx [14–16]. The underlying mechanism through which EVL achieves its antiviral effect is not well known. It has been stated that EVL interacts with the PI3-K pathway [17], an essential protein for viral replication, causing its down-regulation, but it is important to monitor the levels of EVL in this situation, as this drug shows a very narrow therapeutic range [18].

## 4. Conclusion

Our patient had several risk factors for developing CMV infection. First of all, both donor and recipient were CMV positive, received OKT3 as induction therapy, and suffered three rejection episodes during the first year which required use of intravenous steroids. The patient developed the first retinitis while he was receiving CsA + AZA + deflazacort, but the CMV infections were more frequent and serious when MMF was introduced instead of AZA. After this switch, the patient had to receive multiple doses of VGC and foscarnet, and CsA was first reduce and later changed for EVL, but the patient still developed new CMV infections. Finally, MMF was removed and VGC (450 mg/12 h) was introduced as permanent, and the patient has not suffered any infection since that time. Unfortunately, we could not perform the CMV resistance analysis, but we think that in our case the immunosuppressive regimen has been determinant in the infection rate of our patient, especially MMF. That is why we propose, in cases of repeated CMV infection, not only specific treatment against CMV, but also a change in the immunosuppressive regimen.

## Figures and Tables

**Table 1:** 

	2001	2001	2002	2004	2004	2005	2010	2010	2012
Event	HTx	3 rejections	Retinitis	Protocol change	Retinitis	Retinitis	Diarrhea	Retinitis pneumonia	None
Immunosupresion	OKT3 CsA Aza Ster.	Steroids boluses	CsA Aza Steroids	CsA Aza → MMF Steroids	CsA MMF Steroids	↓ CsA ↓ MMF Steroids	Csa → Eve MMF Steroids	Eve Steroids	Eve Steroids
CMV (blood)	R IgG+ D IgG+	Positive Ag.	Negative Ag.	Negative Ag.	Negative Ag.	Positive Ag.	525,000 copies	425,000 copies	Negative
Treatment CMV	None	Ganciclovir oral	Aciclovir iv Aciclovir oral	None	Aciclovir iv VGC	VGC	VGC	VGC Foscarnet iv Foscarnet intraocular	VGC prophylactic

HTx: heart transplantation; CsA: cyclosporine; Aza: azathioprine; MMF: mycophenolate mofetil; Ag: antigenemia; Eve: everolimus; VGC: valganciclovir.
